# Behavioral Insights Into Micronutrient Powder Use for Childhood Anemia in Arequipa, Peru

**DOI:** 10.9745/GHSP-D-20-00078

**Published:** 2020-12-23

**Authors:** Jessica D. Brewer, Julianna Shinnick, Karina Román, Maria P. Santos, Valerie A. Paz-Soldan, Alison M. Buttenheim

**Affiliations:** a Department of Global Community Health and Behavioral Sciences, Tulane University School of Public Health and Tropical Medicine, New Orleans, LA, USA.; b Department of Family and Community Health, University of Pennsylvania School of Nursing, Philadelphia, PA, USA.; c Department of Health Management, Universidad Peruana Cayetano Heredia Facultad de Salud Pública y Administración Carlos Vidal Layseca, Lima, Peru.; d Asociación Benéfica PRISMA, Lima, Peru.; e Zoonotic Disease Research Lab, Universidad Peruana Cayetano Heredia Facultad de Salud Pública y Administración Carlos Vidal Layseca, Arequipa, Peru.; f Center for Health Incentives and Behavioral Economics, University of Pennsylvania, Philadelphia, PA, USA.

## Abstract

Childhood anemia remains a significant driver of morbidity in low- and middle-income countries, including Peru. To identify behavioral challenges to using micronutrient powder (MNP) that is given to supplement children’s diets and prevent anemia, we applied a behavioral design approach to interviews and focus groups with 129 caregivers in Arequipa, Peru. We examined 3 key points in the decision-making process: accessing MNP through the health system; forming intentions to use MNP; and MNP use at the time of child feeding. Using the NUDGE (Narrow, Understand, Discover, Generate, Evaluate) approach, we identified the following behavioral barriers and facilitators: (1) caregivers’ experiences with health care providers shaped their motivation to access MNP; (2) caregivers felt accessing MNP at clinics was inconvenient and created hassle factors; (3) caregivers’ mental models about anemia prevention shaped MNP intentions and use; (4) caregivers’ salient negative experiences could have caused them to stop giving MNP; (5) caregivers forgot to give MNP if they did not have cues to remind them but could be prompted with salient cues; and (6) caregivers were affected by emotional, cognitive, and attentional factors during feeding that were difficult to anticipate. Our results, based on a behavioral design approach, suggest opportunities to adapt current messaging, counseling, and education around MNP use. Adaptations include providing culturally relevant messages, leveraging caregivers’ emotional and cognitive states, and encouraging small but impactful changes to feeding routines to address barriers to MNP use.

## Abstract (precis)

Health care provider-caregiver interactions and caregivers’ shifting emotional states between intention formation and use affected their adherence to a government-provided micronutrient powder intervention to prevent childhood anemia. In counseling directed to caregivers, we suggest providers offer clear messaging on MNP impact and planning for challenges during MNP use.

## Teaser

**Key Findings:**

- Negative experiences with health care providers or inconvenience at the time that micronutrient powder (MNP) use is initiated may discourage future MNP use.
- Mental models about nutrition can shape intentions to use MNP, and having too many choices can confuse caregivers.
- A single negative experience with MNP can form strong memories and discourage caregivers from giving MNP.

**Key Implications:**

- Training for health care providers should encourage positive interpersonal interaction with caregivers during initiation of MNP because these interactions can have a lasting impact on MNP use.
- Education for caregivers should include counseling about potentially challenging side effects so that caregivers are prepared to work through them.
- In future programming, public health practitioners should consider encouraging caretakers to utilize well-timed cues to administer MNP.

Resumen en español al final del artículo.

## INTRODUCTION

Anemia in children can impair cognitive and motor function and cause fatigue and poor school performance.^1^ ^–^ ^2^ It is a significant public health issue, particularly affecting low- and middle-income countries (LMICs).^3^ ^–^ ^4^ In Peru, prevalence of anemia among children aged 6 months to 3 years was 43.6% in 2017.^5^ To combat this high prevalence, in 2014, the Peruvian Ministry of Health began distributing free micronutrient powders (MNPs) (or “chispitas”) to children aged 6 months to 3 years at public health care facilities during well child checkups, where caregivers receive guidance from health care providers on how to apply MNP to children’s meals.^6^ ^–^ ^7^ MNPs are single-dose sachets formulated typically with iron, zinc, folic acid, vitamin A, and vitamin C that are consumed by mixing with semisolid food.^8^ ^–^ ^9^ They are proven to be an efficacious intervention for early childhood anemia, and the World Health Organization recommends their use in populations where the prevalence of early childhood anemia is 20% or higher.^8^ ^,^ ^10^ According to Peru’s national guidelines, caregivers are instructed to use MNP daily for 1 year after beginning the first dose, ideally starting at age 6 months with the introduction of complementary foods.^11^ ^–^ ^12^

Despite these efforts, childhood anemia prevalence in Peru has remained high,^5^ and an early evaluation of national rollout of the MNP program showed low adherence.^7^ Previous research on MNP use in Peru has found that confusion about MNP administration, MNP’s unpleasant taste, side effects, lack of familial and peer support, and negative interactions with those who distribute MNP were barriers to adherence.^7^ ^,^ ^13^ ^–^ ^15^ Alternatively, key facilitators for MNP use were interpersonal support, concern about the long-term effects of anemia, and tailored counseling.^7^ ^,^ ^13^ ^–^ ^15^ Studies on MNP use in other countries confirm that these factors affect MNP program effectiveness.^16^ ^–^ ^23^ Previous research on MNP interventions have led to programmatic changes in other countries such as health care providers including warnings about possible side effects in their counseling to caregivers, recommendations to administer MNP on a flexible instead of fixed schedule, and promotion through educational campaigns with community health providers, among others.^24^ ^–^ ^28^

Despite Peru having national guidelines on MNP use, adherence is low and child anemia prevalence remains high.

### Behavioral Economics and Intervention Design

Several studies have identified social, psychological, and environmental factors that inhibit or enable MNP use; we extend that research here with an applied behavioral design approach, informed by behavioral economics, to understand the behavioral processes at play in MNP use. Behavioral economics—a field that sits at the intersection of economics and psychology—seeks to understand how common mental biases, heuristic thinking, and social forces shape decision making and behavior.^29^ ^–^ ^31^ A rich theoretical and empirical collection of literature from behavioral economics and related disciplines describes and characterizes how decision making often deviates from what rational actor or expected utility models would predict. Consistent findings in this interdisciplinary literature are that humans heavily rely on “rules of thumb” and mental shortcuts to make decisions, are given imperfect information, have time-inconsistent preferences, and have attentional and cognition constraints. These decisions are often not in their best long-term interest but satisfy their immediate needs and desires.

Bringing a behavioral economics perspective to the analysis of uptake of public health programs can help identify specific barriers to and facilitators of target behaviors that are not captured by other approaches. We define behavioral barriers as those factors arising from cognitive or psychological processes that reduce the likelihood of a target behavior being carried out; behavioral facilitators similarly increase that likelihood.^32^ ^–^ ^34^ Behavioral barriers and facilitators often operate separately from conscious cognition or awareness; one implication of this is that people’s statements about their intentions, motivations, and decisions around a behavior—particularly a complex or habitual behavior—may paint an incomplete picture of that behavior’s context. In recent years, innovative methods have emerged that map contextual data about a behavior (including field observations; interviews and focus groups with participants, stakeholders, and experts; and existing quantitative evidence and prior literature) to specific behavioral economics principles to uncover novel insights about barriers and facilitators that can inform intervention design. These methods and approaches have been widely used in a variety of global health settings and programmatic domains^32^ ^,^ ^33^ ^,^ ^35^ ^,^ ^36^ and in previous work on food choices and human nutrition.^37^ ^–^ ^40^

Because the provision of MNP is an active intervention that requires multiple steps, sustained action over time, and the translation of intentions into behavior, behavioral economics may offer novel insights into low adherence to MNP use despite its availability and promotion. Interventions informed by behavioral economics have been used successfully in prior studies to improve maternal and child nutrition, from simple changes to the layout of school cafeterias and providing verbal cues for healthier choices in the United States,^41^ ^–^ ^42^ to incentives and reminders to buy healthy foods in Madagascar that are designed to address specific behavioral barriers (for example, incentives to address procrastination and present-orientation, and stickers that deliver salient reminders at the point of purchase and consumption.).^43^

Behavioral economics analysis may offer novel insights into low adherence to MNP use despite its availability and promotion.

In this article, we advance our understanding of behavioral barriers to and facilitators of consistent MNP use for anemia prevention in Peru. Using previously collected contextual inquiry data, we applied a behavioral design approach to uncover novel insights about caregiver choices and actions related to giving MNP. These insights can inform counseling techniques used in MNP programs.

## METHODS

### Setting

We conducted our study in Arequipa, the second largest city in Peru, which has particularly high rates of childhood anemia.^5^ Despite ongoing efforts to address anemia, 44.5% of children aged 6 months to 3 years in Arequipa were diagnosed with anemia in 2016.^44^ The study was conducted in 8 of 29 districts in Arequipa, which accounted for more than half of the cases of early childhood anemia in the province according to unpublished sources from the local branch of the Ministry of Health.

### Data

In 2017, we conducted 24 interviews and 12 focus groups with caregivers of children aged 6 months to 3 years. Caregivers were defined as adults who self-reported spending at least 5 days a week providing care for the child, whether they were the child’s biological parent or otherwise. This inclusion criterion was established as the only one for recruiting caregivers as we believe the primary caregiver is typically in charge of child feeding and thus the administration, or lack thereof, of MNP and so that we could obtain a range of caregiver experiences related to gender, caregiver age, child age, child history of anemia, caregiver-child relationship, and other factors. Caregivers were selected for interviews via convenience sampling in and around local health establishments and selected for focus groups through door-to-door recruitment in the neighborhoods surrounding the health establishment. The interview and focus group guides (Supplement 1) were developed to probe for caregiver experiences in obtaining MNP from health care providers and applying MNP to children’s meals, as well as other beliefs about anemia treatment and prevention. Interviews and focus groups were conducted by authors JDB, KR, and MPS in Spanish, audio recorded, and transcribed in Spanish. In total, we conducted individual interviews with 24 caregivers and 12 focus groups with 4 to 13 caregivers each, resulting in a total of 129 caregiver participants. The study team analyzed a subset of data from this parent study for the present analysis. More details about the data from the parent study are available from Brewer et al.^15^

### Analytical Approach

We used the NUDGE (Narrow, Understand, Discover, Generate, Evaluate) approach to analyze behavioral barriers to and facilitators of MNP use among caregivers. NUDGE was developed to support the systematic and rigorous application of behavioral economics insights to intervention design,^45^ and is one of several published design approaches informed by behavioral economics and design thinking.^33^ ^,^ ^46^ The use of the term “nudge” is intentional; the approach generates intervention designs that are consistent with Sunstein and Thaler’s definition of nudges^31^:

*any aspect of the choice architecture that alters people's behavior in a predictable way without forbidding any options or significantly changing their economic incentives.*

NUDGE includes 5 stages:

1. Narrow the focus of the analysis to a specific, relevant behavioral target
2. Understand the context of the behavior through inquiry into the decision-making process and related actions
3. Discover insights about barriers to and facilitators of the target behavior through structured matching of elements from contextual understanding developed in Stage 2 to core principles (cognitive biases and heuristic thinking) from behavioral economics
4. Generate intervention strategies and designs to address identified barriers
5. Evaluate those designs through iterative prototyping and trialing

In this article, we report the results from the Narrow, Understand, and Discover stages (Figure).

**FIGURE:**
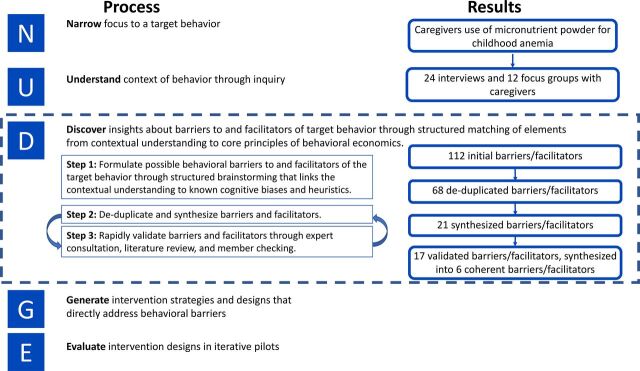
NUDGE Approach as Applied to Analyzing Behavioral Barriers to and Facilitators of Micronutrient Powder Use Among Caregivers Abbreviation: NUDGE, narrow, understand, discover, generate, evaluate.

Building on a previous analysis utilizing the social-ecological model to identify factors that inhibited and enabled MNP use from Brewer et al.,^15^ we narrowed our point of inquiry to a defined behavioral target: the regular use of MNP during child feeding. We developed a rich understanding of the context around MNP use through repeated reading of focus group and interview data. To discover relevant behavioral insights, we first identified the key decisions and actions underlying the target behavior. Next, using a set of prompts about the cues, meanings, and alternatives related to decision and action steps, we brainstormed barriers to or facilitators of each step. Each barrier linked the contextual understanding developed in the previous stage with 1 or more specific behavioral economics constructs (e.g., availability heuristic or present bias) to discover an insight about barriers and facilitators for the target behavior. Three examples of how a barrier or facilitator is discovered from contextual inquiry and behavioral constructs are shown in the Table.

**TABLE.** Summary of Discovery of Behavioral Barriers/Facilitators to Micronutrient Powder Use Among Caregivers in Peru

**Decision-making Step**	**Prompt** **(Cue, Action, or Meaning)**	**Contextual Factor**	**Behavioral Construct**	**Barrier or Facilitator**
Accessing MNP	Cue: The perspectives of people with authority on MNP hold greater weight	Caregivers are more likely to make decisions about their children’s health when an expert gives them the information.	Authority bias	Negative interactions with medical professionals can cause caregivers to not give MNP.
Using MNP at the moment of child feeding	Meaning: Is the action uncomfortable or painful such that it is avoided?	Children often react negatively to taste of MNP, making feeding difficult or unpleasant for caregiver.	Negativity bias	If children refuse food supplemented with MNP, caregivers may stop giving it.
Using MNP at the moment of child feeding	Action: Caregivers abruptly discontinue MNP	Caregivers abruptly decide to stop giving MNP when their child has diarrhea or another side effect.	Hot-to-cold empathy gap	Visceral reactions when a child is sick can lead to a rapid choice to discontinue MNP.

Abbreviation: MNP, micronutrient powder.

This process yielded 121 barriers and facilitators related to MNP use, which were de-duplicated to 68, synthesized to 21, and validated in an iterative process to ensure rigor and completeness. The validation process included in-depth discussions with authors KR and MPS, who are public health researchers involved in original data collection but who had not previously participated in this analytic process. This validation process resulted in the exclusion of 4 barriers (and related behavioral constructs) that did not align with the researchers’ experiences in the field. The validated list of 17 barriers and facilitators were further reduced to a set of 6 coherent, validated barriers to and facilitators of the target behavior that can inform intervention design. Importantly, the results of this analytic process are at the level of the barrier to or facilitator of the focal behavior; while each barrier/facilitator is informed by 1 or more behavioral constructs, the behavioral constructs themselves are not the results of the analysis.

## RESULTS

We identified 6 behavioral barriers to and facilitators of MNP use that operate at 3 key points along the intention-to-behavior continuum: (1) accessing MNP through the health system, (2) forming an intention to use MNP, and (3) using MNP at the moment of child feeding. Examples of qualitative data illustrative of and supporting the behavioral constructs underlying each barrier/facilitator are available in Supplement 2.

### Accessing MNP Through the Health System

#### 1. Caregivers’ Experiences With Health Care Providers Shaped Their Motivation to Access MNP

Caregivers accessed MNP and received counseling about its use at health clinics during well child checkups. Because health care providers were seen as authority figures, the emotional valence of these interactions was influential. Some caregivers reported that negative interactions with health care providers (e.g., feeling dismissed, condescended to, rushed, shamed) made them reluctant to return to health establishments. Negative interactions may have also dissuaded caregivers from taking the advice about MNP given by that professional at the visit. Unclear or contradictory information from health care providers about MNP and its use could have led to ambiguity aversion. Some caregivers avoided using MNP if they felt they lacked sufficient information about it, especially if they were unsure about its potential harms. They expressed uncertainty about both effectiveness and administration of MNP. We originally posited that caregivers might also have avoided asking health care providers questions to resolve these doubts about MNP due to social desirability bias or wanting to appear competent in front of health care providers, but this was not supported during the validation step based on observations made during data collection.

Unclear or contradictory information from providers about MNP could have led to ambiguity aversion.

In other circumstances, interactions with authority figures could have also acted as facilitators for MNP use. For example, sometimes it was easier for caregivers to give MNP if they felt like someone else (health care providers, family members) told them they had to give MNP, essentially making the decision for them. However, it should be noted that a few caregivers were uncomfortable with health care providers presenting MNP as a requirement, especially if they already doubted its quality or felt like they had not been given explanations for the reasons to use it. Additionally, caregivers who felt they had received good information from authority figures felt more confident about using MNP. For example, caregivers expressed satisfaction when health care providers took the time to explain MNP to them in depth and address their doubts. Finally, framing effects (how health care providers framed anemia to caregivers) affected their likelihood of administering MNP. For example, caregivers reported they were more likely to give MNP if they were told how anemia could affect their child’s brain and development, whereas being told their child had “low hemoglobin” was confusing and did not instill a sense of urgency.

Caregivers were more likely to give MNP if they were told how anemia could affect their child’s brain and development.

#### 2. Caregivers Felt Accessing MNP at Clinics Was Inconvenient and Created Hassle Factors

Even the smallest amount of friction or hassle reduced the probability that caregivers would access MNP or seek information on its use. If caregivers were required to attend informational sessions at inconvenient times or knew they would have to wait in long lines for a well child checkup (where they received MNP and counseling), they may have been reluctant to make the visit. We originally also posited that once caregivers already had MNP at home, they were more likely to give it to their child, an example of endowment effect, a psychological phenomenon where people are more likely to keep something they already have than make the effort to obtain it. However, this effect was not validated by other researchers given that caregivers were given an exact amount of MNP to last them between checkups (approximately 90 sachets) and thus would not have had any extra that would allow for prolonged use. Losing MNP sachets or other problems related to the number of sachets received was not a salient theme in our analysis.

### Forming an Intention to Use MNP

#### 3. Caregivers’ Mental Models About Anemia Prevention Shaped MNP Intentions and Use

Caregivers’ mental models about nutrition, how MNP works, and what could serve as a substitute for MNP directly shaped intentions to use MNP. Caregivers who perceived multiple ways to treat or prevent anemia, such as a variety of over-the-counter medications, may have experienced choice overload and may have looked for simplifying heuristics to choose among known alternatives, such as salience, familiarity, or ease. Some caregivers expressed the belief that over-the-counter medications were of higher quality than MNP, given that they were distributed by pharmacies (instead of the public health system) and had a monetary cost (as opposed to free distribution). Caregivers’ preferences to treat anemia through diet reflected the mental model (sustained primarily by peer or family advice but also recommended by health care providers) that a “natural” solution was preferable to a pharmacological one. Additionally, caregivers often defaulted to the use of food, a traditional and automatic response to treating illness, prompted by its presence in the home. A few caregivers expressed distrust of MNP because of its manufacture in India, which we hypothesized may have reflected a possible “not invented here” bias that may have limited regular use. During the validation process, other researchers concluded that this barrier may not have been as salient as other heuristics given its less frequent occurrence in the data. However, other mental models that drew analogies between MNP and something more familiar could have facilitated MNP use. Caregivers who described MNP as being “like vitamins” (versus medication) appeared more likely to have favorable views of MNP and to feel comfortable using it.

Caregivers’ preferences to treat anemia through diet reflected their preference for a “natural” solution over a pharmacological one.

#### 4. Caregivers’ Salient Negative Experiences Could Have Caused Them to Stop Giving MNP

Caregivers accumulated positive and negative experiences, both personal and secondhand, about MNP. However, negativity bias led them to pay attention to and remember the negative experiences more. For example, caregivers who experienced frustrations with MNP use in the past, due to the child experiencing side effects like diarrhea or refusing to eat foods with MNP because of its taste, may have lost their intention to continue to use MNP. Negative side effects like diarrhea were immediately evident to caregivers and remained in their memories, compared to positive effects like higher hemoglobin levels which were invisible to the caregiver and had more gradual, long-term effects on the child’s health. Anecdotal fallacy and base rate neglect also occurred when caregivers gave higher weight to a few salient stories from their peers about troublesome MNP side effects, as opposed to following medical advice from health care professionals. This over-anchoring on negative effects reduced intentions to use MNP going forward. During the validation phase, other researchers agreed that caregivers disproportionately focused on the negative effects of MNP, especially related to side effects and taste.

### Using MNP at the Moment of Child Feeding

#### 5. Caregivers Forgot to Give MNP if They Did Not Have Cues to Remind Them but Could Be Prompted With Salient Cues

Even when individuals intend to do something and have the resources to do it, they often require a specific prompt from the environment to overcome inertia. If the social, physical, or media environment failed to cue MNP use at the right time and in the right way, caregivers may have defaulted to nonuse. For example, some caregivers did not feel prompted to use MNP if their child appeared to be healthy, even if the child had been diagnosed with anemia. It may have been possible that they experienced “ostrich effect,” or unwillingness to accept this diagnosis for fear of dealing with the repercussions. The authors involved in data analysis originally posited that if caregivers did not see others in their communities using MNP, they may have assumed that others did not approve of MNP or simply that not seeing peers use MNP could have failed to cue MNP use. This was not supported during validation. Another early proposed barrier was that working or busy caregivers who bought prepared food instead of cooking homemade meals were less likely to add MNP to the purchased food because they did not go through the process of preparing and serving the food themselves (cue-dependent forgetting); however, this was eliminated during validation given that the relevant data referred to general nutrition practices, not MNP use. Alternatively, salient, well-timed cues from the environment could have promoted MNP use. Caregivers strongly suggested that additional information about MNP on mass media, particularly television and radio, could have been an effective cue.

If the social, physical, or media environment failed to cue MNP use at the right time and in the right way, caregivers may have defaulted to nonuse.

#### 6. Caregivers Were Affected by Emotional, Cognitive, and Attentional Factors During Feeding That Were Difficult to Anticipate

The challenges of using MNP in the moment during child feeding could have led to procrastination and avoidance, exacerbated by the hot-cold empathy gap. When caregivers were at the clinic and decided to use MNP, they were in a deliberative and rational or “cold” state. This made it difficult to envision what it would have been like to apply MNP at mealtime, when caregivers were in an agitated, cognitively taxed “hot state” due to child experiences with side effects or dislike of taste. The counseling that caregivers received at well child checkups did not acknowledge this gap or help caregivers plan for it. In the validation process, other researchers confirmed that aversion to MNP at the moment of use led to its avoidance, especially if caregivers did not feel prepared to address any complications that may arise.

Some caregivers also over-focused on specific details of MNP administration, known as focusing effect, which made MNP easy to abandon if they felt they could not perfectly follow the instructions. Because skipping or incorrectly implementing a step may have resulted in worse taste or side effects (e.g., leaving it in food for an extended period of time increased the metallic taste due to capsule breakdown), this focus was understandable, but it may have led to abandoning MNP administration in the moment after a minor deviation from the protocol. Alternatively, when caregivers were removed from the process of administering MNP to the child, they expressed greater satisfaction with MNP and its effect on their child’s health. This was the case for caregivers who used Cuna Mas, a public daycare that required them to bring in MNP with their child so the staff could administer it to the child during the day.

## DISCUSSION

We identified 6 behavioral barriers to and facilitators of using MNP for anemia prevention among caregivers of young children in Arequipa, Peru. These are: (1) caregivers’ experiences with health care providers shaped their motivation to access MNP; (2) caregivers felt accessing MNP at clinics was inconvenient and created hassle factors; (3) caregivers’ mental models about anemia prevention shaped MNP intentions and use; (4) caregivers’ salient negative experiences could have caused them to stop giving MNP; (5) caregivers forgot to give MNP if they did not have cues to remind them, but could be prompted with salient cues; and (6) caregivers were affected by emotional, cognitive, and attentional factors during feeding that were difficult to anticipate. Our results support and extend previous findings in the literature and provide opportunities for designing and revising program interventions that incorporate the behavioral constructs underlying the barriers.

First, as in other studies, we found that authority figures including health care providers have an opportunity to influence uptake and ongoing use of MNP.^7^ ^,^ ^13^ ^,^ ^17^ ^,^ ^19^ Our results place unique emphasis on the emotional context of these interactions. Caregivers had different perspectives on what constituted a positive or negative interaction with health care providers; some preferred an authoritative approach and others preferred collaborative decision making. Although research in Peru and many other countries promotes the use of culturally appropriate counseling techniques,^13^ ^,^ ^17^ ^,^ ^26^ research on collaborative decision making between health care providers and patients regarding nutrition is limited to the United States.^47^ Future research could examine whether authoritative or collaborative counseling styles would be most effective in motivating caregivers in Peru. In addition, as in other studies, we found that confusing information during consultations could have led to caregivers feeling they lacked sufficient information on MNP; this reinforces the need to simplify and tailor the educational campaigns recommended by other studies.^13^ ^,^ ^19^ ^,^ ^26^ Our results also highlight that framing anemia as “low hemoglobin” reduced both salience and urgency for caregivers; a higher salience framing in Ministry of Health campaign and health care provider training materials could emphasize children’s growth and brain development.

Giving caregivers confusing information during visits could have led to them feeling they lacked sufficient information on MNP—reinforcing the need to simplify and tailor educational campaigns.

Second, our study highlights hassle factors as another major barrier when accessing MNP at clinics. Although prior studies have identified barriers to accessing health services, few connect those barriers to MNP adherence. Prior behavior science research has demonstrated that even minimal friction in a health or benefits program reduced take-up.^46^ In the context of MNP use, hassle factors reduced caregivers’ likelihood of accessing MNP and receiving information on its use. Therefore, our results point to the need for structural changes within the health system (more staff available for appointments, creating appointment and informational session schedules outside of caregivers’ work hours, and increasing access to MNP sachet “refills” in community settings). Cuna Más, a public daycare system in Peru, and community health promoters are trusted sources that could expand access to MNP.

Our third and fourth results concerned forming an intention to use MNP that we demonstrated was shaped by mental models about nutrition and by negative experiences with MNP. Caregivers may have had a preference for addressing anemia through diet, a more familiar and less “medicalized” approach than MNP. This default preference for dietary approaches may have led caregivers to attend to and implement dietary suggestions rather than use MNP even when health care providers made both recommendations. Our results also revealed that a way to frame MNP to align with the caregiver mental models about nutrition was as a vitamin supplement rather than as a medication. Negative experiences with MNP, such as side effects or bad taste, and the effect of negative comments from family and peers, have all been noted in previous studies.^7^ ^,^ ^13^ ^,^ ^15^ ^,^ ^17^ ^–^ ^19^ ^,^ ^22^ Although previous work has typically interpreted negative comments as lack of social support for MNP use and therefore proposed increased informational outreach to family members and peers as a solution,^17^ our results highlight the importance of including specific behavioral guidance to family and peers to not to overemphasize prior complications and negative experiences.

For any behavior that is new, challenging, and not yet habitual, it’s easy to procrastinate. In our fifth result, we confirmed a common finding from previous studies that caregivers needed external cues to overcome procrastination around MNP use at mealtime. General prompts in the form of television and radio spots were useful, and they have been used in Peru by Ministry of Health and other organizations to effectively promote anemia-specific^48^ and other positive health behaviors.^49^ ^–^ ^50^ Our approach also uncovered the importance of specific, timely, unavoidable cues at the moment of child feeding, which may drive behavior change more than a TV or radio spot heard earlier in the day. Possible interventions informed by this insight include encouraging caregivers to store MNP sachets with other items that will be used during mealtime (i.e., with the child’s dish or utensils) or sending an SMS message reminder at common mealtimes.

Finally, we identified “hot-cold empathy gap” as a barrier to consistent MNP use. Hot-cold empathy gap has been observed in other health behaviors, in which people consistently fail to imagine and account for what a future “hot” affective or cognitive state will be when a plan to act is made ahead of time in a “cold” state.^51^ ^–^ ^54^ Hot-cold empathy gap is particularly relevant for understanding what happened when caregivers intended or planned to use MNP during child feeding but abandoned that plan in the moment when children resisted, refused, reacted strongly to taste, or experienced side effects such as diarrhea, all of which are commonly cited barriers in the literature.^7^ ^,^ ^13^ ^,^ ^15^ ^,^ ^18^ ^,^ ^19^ ^,^ ^55^ ^–^ ^59^ Awareness of the hot-cold empathy gap can provide a channel for improved program development. Given that people generally don’t understand their actions as “state-dependent,” a possible intervention involves preparing caregivers for side effects that might cause anxiety. Health care professionals could encourage caregivers to think through how they would react to a stressful experience while in a “cold state” at their medical appointment. This strategy is based on evidence from the side effect reduction literature, often focused on cancer patients, that suggests that preparation for side effects can reduce anticipatory symptoms and stress and improve coping skills.^60^ ^–^ ^61^ This approach would represent a departure from current Peruvian Ministry of Health trainings that teach health care providers to counsel caregivers that MNP has no side effects.^13^

The hot-cold empathy gap helps us understand what happened when caregivers planned to use MNP but abandoned that plan when they experienced barriers such as a child’s negative reaction.

### Limitations

There are several limitations to this study. Brewer et al. describes the limitations in study design and data collection, such as recruitment of participants in and around health centers, the reliance on self-reported (rather than observed) barriers to MNP use, and the lack of systematic collection of sociodemographic and behavioral characteristics of the caregivers and their children (such as time using MNP, birth order, etc.).^15^ Adopting a behavioral economics perspective limits the identified barriers and facilitators to those with a specific behavioral (as opposed to structural) underpinning. Additionally, the behavioral barriers to optimal MNP use identified in this analysis require further confirmation through empirical testing of interventions designed to address them. Although NUDGE is similar to several other approaches using behavioral economics and human-centered design, it is still evolving as an analytic tool and future iterations may further refine the approach.

## CONCLUSION

This study uses behavioral economics and a behavioral design approach to understand MNP administration and childhood anemia prevention generally. This approach to analyzing cognitive biases and heuristics can generate insights into behavioral influences on adherence that complement existing approaches to identifying barriers to take-up of evidence-based practices. Our results led us to focus on various underlying heuristics that influenced MNP adherence, such as authority bias and framing effects, hassle factors, the salience of negative experiences and certain mental models, appropriately timed cues, and the hot-cold empathy gap. Consideration of these behaviors and underlying biases may inform aspects of programmatic intervention, such as counseling practices to promote MNP use, to improve adherence in an area of Peru that has a disproportionately high burden of anemia.

## Supplementary Material

20-00078-Brewer-Supplement2.pdf

20-00078-Brewer-Supplement1.pdf

## Notes

**En español**

**Perspectivas Conductuales Sobre el Uso de Micronutrientes en Polvo Para la Anemia Infantil en Arequipa, Perú**

**Mensaje clave**: Las interacciones entre el personal de salud y los cuidadores de niños y sus estados emocionales cambiantes, desde que forman la intención para usar los micronutrientes en polvo (MNP) hasta que los usan afectaron su adherencia a los MNP, proporcionados por el gobierno para prevenir la anemia infantil. Durante la consejería para cuidadores, sugerimos que el personal de salud proporcione mensajes claros sobre el impacto de los MNP y una estrategia para enfrentar los retos que podrían encontrar en el uso de los MNP.

**Hallazgos claves:**

- Experiencias negativas con el personal de salud o inconveniencias al momento que se inicia el uso de los MNP pueden desalentar el uso futuro de los MNP.
- Modelos mentales sobre la nutrición pueden influir en las intenciones para usar los MNP y, a la vez, el hechos de tener demasiadas opciones puede confundir a los cuidadores.
- Una sola experiencia negativa con los MNP puede formar memorias impactantes y desalentar a los cuidadores a dar los MNP.

**Implicaciones claves:**

- La capacitación para el personal de salud debería fomentar una interacción interpersonal positiva con los cuidadores durante la iniciación de los MNP porque estas interacciones pueden tener un impacto duradero en el uso de los MNP.
- La consejería para cuidadores debería incluir información sobre efectos secundarios que podrían tener los niños para estar preparados para manejarlos.
- En programación futura, los profesionales de la salud pública deben considerar alentar a los cuidadores a utilizar señales oportunas para administrar MNP.

**Resumen**

La anemia infantil sigue siendo un factor importante de morbilidad en los países de ingresos bajos y medios, incluyendo al Perú. Para identificar los retos conductuales para el uso de los micronutrientes en polvo (MNP), administrados con el fin de complementar la alimentación de los niños y prevenir la anemia, aplicamos un enfoque del diseño del comportamiento (*behavioral design*) en entrevistas y grupos focales realizadas con 129 cuidadores en Arequipa, Perú. Examinamos 3 puntos claves en el proceso de toma de decisiones: acceso a los MNP a través del sistema de salud; formación de la intención para usar los MNP; y el uso de los MNP al momento de alimentar al niño. Utilizando la estrategia de NUDGE (de las siglas en inglés para Reducir, Entender, Descubrir, Generar y Evaluar), identificamos las siguientes barreras y facilitadoras de comportamiento: (1)Las experiencias de los cuidadores con el personal de salud determinaron su motivación para acceder a los MNP; (2) Los cuidadores sintieron que acceder a los MNP en las clínicas era inconveniente y creaba molestias (*hassle factors*); (3) Los modelos mentales de los cuidadores sobre la prevención de anemia influyeron en la intención y el uso de los MNP; (4) Una experiencia negativa marcada de los cuidadores pudo haber hecho que dejaran de dar los MNP; (5) Los cuidadores se olvidaban de dar los MNP si no tenían señales (*cues*) para recordárselos, pero podían recibir señales oportunas para su administración; y (6) Los cuidadores fueron afectados durante la alimentación por factores emocionales, cognitivos y de atención, que fueron difíciles de anticipar. Nuestros resultados, basados en un enfoque del diseño del comportamiento, sugieren oportunidades de adaptar la comunicación, orientación y educación actual sobre el uso de los MNP. Las modificaciones incluyen proporcionar una comunicación culturalmente relevante, aprovechando los estados emocionales y cognitivos de los cuidadores, y alentar cambios pequeños pero impactantes en la rutina de alimentación para combatir las barreras al uso de los MNP.

## Notes

**Peer Reviewed**

**First published online:** December 3, 2020

**Funding:** This work was supported by the National Institute on Minority Health and Health Disparities, “Tulane-Xavier Minority Training in International Health” through the Minority Health International Research Training Program [grant number T37 MD001424]; the Stone Center for Latin American Studies at Tulane University with the Tinker Foundation; and the Fulbright U.S. Student Program. The conclusions expressed in this article are entirely those of the listed authors and are not endorsed by the Fulbright Program, the U.S. Department of State, or any of its partner organizations. None of the funders had any role in study design, data collection and analysis, decision to publish, or manuscript preparation.

**Author contributions** KR and VPS were involved in study design and administration. Data was collected by JDB, KR, and MPS. Analysis was conducted by JDB, JS, AB, KR, and MPS. JDB, JS, and AB prepared the manuscript, and KR, MPS, and VPS provided input and edited various drafts.
