# Supplementary material for: Behavioral Insights Into Micronutrient Powder Use for Childhood Anemia in Arequipa, Peru
Source: Glob Health Sci Pract. 2020 Dec 23;8(4):721–31. doi: 10.9745/GHSP-D-20-00078 (PMC7784068; doi:10.9745/GHSP-D-20-00078)
Supplement: 20-00078-Brewer-Supplement2.pdf [file 20-00078-Brewer-Supplement2.pdf]

Supplement to: Brewer JD, Shinnick J, Román K, Santos MP, Paz-Soldan VA, Buttenheim AM. Behavioral economic insights into micronutrient powder use for childhood anemia in Arequipa, Peru. *Glob Health Sci Pract*. 2020;8(4). https://doi.org/10.9745/GHSP-D-20-00078

Supplement 2. Examples of Qualitative Data From Interview Transcripts and Corresponding Behavioral Economic Heuristics or Biases about Micronutrient Powders Among Caregivers in Peru

Barrier or facilitator	Relevant behavioral constructs	Illustrative quotation from interviews and focus groups
**1. Caregivers' motivation to access MNP is shaped by their experiences with healthcare providers.**	Authority bias-negative interactions	Honestly, I've encountered that the doctors are a little bitter. [They say,] "What is your child sick with? It's this. Oh, give this medication. OK, bye."
	Authority bias (positive)	The good thing about the health post is that when my child first got anemia, the doctors were there supporting me. "You have to be there with the baby, helping them. You need to give them this and this." They sent me to the nutritionist; they even went to my house. It was a really nice gesture.
	Ambiguity aversion	I'm going to be sincere. Since I don't have full knowledge about *Chispitas*, what it is, what it contains… I preferred not to give it to my daughter, because I want to know what it really is. For someone to explain to me what it's made out of, who made it. I don't know much, so I left it there, I haven't given it [to her].
	Framing effect	**Have any of you given your children *Chispitas*?**<br>Yes, since he was 6 months old and began to eat. Obviously, he doesn't like them, but I try to give them to him… He had hemoglobin of 11, [they told me], "his hemoglobin is low" and it's worrisome because they keep telling you that yes, it's going to affect his brain. And as a mother, you worry about their growth.
**2. Accessing MNP at clinics is inconvenient and creates hassle factors**	Hassle factors	**And do a lot of moms go? How was [the informational session]?**<br>No, at times, [the moms] don't give it much value, much interest. ... They're not interested. I think it's because of our own laziness.

**3. Caregivers' mental models about anemia prevention shape MNP intentions and use**	Choice overload	**Besides diet, did they give [the child] any treatment or medication?** Those *Chispitas* that you add to their food… Then they prescribed ferrous sulfate to me, or otherwise that I should buy *Emulsión Scott*. "You're also going to give that [to the child]," they told me. I'm treating [the child] with that right now and I hope he gets better.
	Default to food	**And is there something you all have heard of from your neighbors or family members that can cure someone with anemia? Something that wasn't from the health post? Like some sort of tradition?** Giving liver. Diet, more than anything. I think that they don't accept the supplement they give out here; they opt for other things that are natural. Like liver, cooked blood.
	Mental models- MNP as vitamins	**Has anyone else been given ferrous sulfate or anything else for anemia?** They have given us *Chispitas.* **Why did they give those to you?** To strengthen [the child] is what they told me. **Did [the child] have anemia?** No, but [the child] always has to take [MNP] because they are vitamins to make them strong, to avoid anemia. That's what they told me.
**4. A salient negative experience can cause caregivers to stop giving MNP**	Negativity bias	Participant 1: It smells like iron. Participant 2: It has the flavor of iron. Participant 3: Since it has a lot of iron, the food smells like it and it changes the flavor and [the child] no longer wants it. He notices the flavor.
	Anecdotal fallacy and base rate neglect	**Have you talked with a family member or neighbor about *Chispitas* or anemia?** Participant 1: Yes, with almost everyone I've talked to [their child] gets constipated, [another member of the focus group] is the first person who has told me [their child didn't get constipated]. But I have tried giving fruits and juices, and then it helps. But if it doesn't help, [the child] gets constipated.

**5. Caregivers forget to give MNP if they do not have cues to remind them, but can be prompted with salient cues.**	Fails to cue MNP use	An ad has come on TV a lot recently. Before I didn't hear anything... Now that my son was recently born and they are making me monitor him, recently I have heard about anemia.
	Ostrich effect	There are babies like that. There really are babies that are skinny and really have a good [immune system]; and there are babies who are fat and you say, "But how is this baby going to have anemia?" And in reality, they have anemia.
	Salient, well-timed cues	**Have you seen that as well? The television ad?**<br>Participant 1: Yes.<br>**And what did you think about the ad? Did you like it or…?**<br>Participant 2: Well, it made me worried, because I didn't know… In the ad, they say that [anemia] affects the brain… and that worried me.
**6. Caregivers are affected by emotional, cognitive, and attentional factors during feeding that are difficult to anticipate.**	Hot-to-cold empathy gap	**How do they give out *Chispitas* here?**<br>Participant 1: In the Well-Child check up every month… But we don't give them [to our child] because they cause diarrhea.<br>**How long did you use *Chispitas*?**<br>Participant 1: Three months.<br>**And after that?**<br>Participant 1: I didn't after that because they caused so much diarrhea.
	Focusing effect	**And these micronutrients, how are they? What are they made of or how do you give them to kids?**<br>Participant 2: It contains zinc, no?<br>Participant 1: Yes.<br>Participant 2: Yes, it contains zinc, it turns dark.<br>Participant 3: It turns dark and gives off a flavor, like metal.<br>Participant 2: Of course. You have to give it in a little [food], I don't know, like two tablespoons. You give it to [the child], they eat it. If you take a long time giving it to them, that's how it gets. You have to give it quickly.

	Procrastination and avoidance	If children eat something and it doesn't taste good, they're not going to eat it… She doesn't eat [MNP] easily. She notices the taste quickly. So what do I have to do if I want her to eat [her food] too? What do I do? I stopped giving her [MNP] and she eats.
