# Supplementary material for: Behavioral Insights Into Micronutrient Powder Use for Childhood Anemia in Arequipa, Peru
Source: Glob Health Sci Pract. 2020 Dec 23;8(4):721–31. doi: 10.9745/GHSP-D-20-00078 (PMC7784068; doi:10.9745/GHSP-D-20-00078)
Supplement: 20-00078-Brewer-Supplement1.pdf [file 20-00078-Brewer-Supplement1.pdf]

Supplement to: Brewer JD, Shinnick J, Román K, Santos MP, Paz-Soldan VA, Buttenheim AM. Behavioral economic insights into micronutrient powder use for childhood anemia in Arequipa, Peru. *Glob Health Sci Pract*. 2020;8(4). https://doi.org/10.9745/GHSP-D-20-00078

Supplement 2. Focus Group and Participant Interview Guide about Caregiver MNP Use in Peru

**Theme**	Questions/points
**Perception and knowledge/Sources of information about anemia**	
	What is anemia to you?<br>How did you find out about anemia?<br>Do you have any experience with anemia?<br>Do you know what causes anemia?<br>Do you know what the consequences of anemia are?<br>Do you believe that anemia is important or not?<br>Is there anything that can be done to prevent and/or cure anemia?<br>The people in your community, your neighbors, your friends, are they worried about anemia?<br>What do people say about anemia?<br>Is anemia different in adults and children?<br>Do you feel that your children are at risk of having anemia? Why?<br>Were there talks or campaigns here about anemia?<br>During the child's check-ups, did anyone talk to you about anemia? Who? What did they say to you?<br>Are there any or do you know of any stories about anemia here? (Tales, legends, or beliefs?)
**Preventive practices**	
• Personal	Are you doing anything to prevent anemia in your children? What?<br>Are there any other actions to prevent it? Which ones do you know?<br>Are these actions different for adults than for children?<br>What do your friends or other people in your family do to prevent it?
• External or governmental	Does the health system recommend anything to prevent anemia? What do they recommend?<br>Do you do what they say? Why?<br>What do people think about these preventive actions? Do they utilize them? Why?<br>Were there any talks or campaigns here about preventive practices? Have you been to any of them?<br>During the child's check-ups, did anyone talk to you about preventive practices for anemia?
**"Chispitas"/Iron supplements**	
• Use of MNP or "Chispitas"/iron supplements	Do you currently or have you in the past used iron supplements or "Chispitas"?

Supplement to: Brewer JD, Shinnick J, Román K, Santos MP, Paz-Soldan VA, Buttenheim AM. Behavioral economic insights into micronutrient powder use for childhood anemia in Arequipa, Peru. *Glob Health Sci Pract*. 2020;8(4). https://doi.org/10.9745/GHSP-D-20-00078

**Theme**	Questions/points
	How do you know about the existence of these supplements?<br>Do you know how they help or what they are good for?
• Method of preparation (administration, dosage, frequency)	Is it easy to administer them to your child?<br>Can you show me how you go about the process of preparing them?<br>What dosage and with what frequency do you give them to your child?
• Community perception of the product	What do people say about the supplements or "Chispitas"?<br>Do they use them? Why?<br>Would you recommend the supplements to your family and friends?<br>In your opinion, do they think that the supplements are being administrated correctly in homes? Why?<br>Do you think that MNP get to everyone who needs them? Why?
• Perception of benefits	Do you believe that MNP are beneficial for your child? Why?
• Perception of risks	Do you believe that your child has shown a reaction from consuming food with MNP? Why do you think this is related to MNP? Was it a good or bad reaction?
**Important elements in accordance with the utilized theory**	
• Microsystem	Which person in your family do you believe has the most influence on your way of thinking or in the moment of making a decision?<br>Have they ever changed your way of thinking after you had made a decision?
• Mesosystem	Your parents, what do they think about MNP? Do they advise you to give MNP to you son(s)/daughter(s)?<br>Have you ever spoken with them about MNP? What do they think?<br>Have you ever spoken with your friends about MNP? What do they think? Do they give their children MNP?
• Exosystem	How would you grade the counseling from the CRED service about MNP? Why? How would you have liked your counseling about MNP to have been? Do you see any change in your child due to administration of MNP? What changes? What would you like to be changed about the care of your child in CRED service for MNP?
• Macrosystem	Is there any sign inside the health establishment that is focused on MNP? In the CRED office, did they provide you any publicity material about the program? Is the environment in CRED private, exclusive for this purpose? If you could grade the care from the CRED service in regards to MNP on a scale of 1-10, what number would you give them? What do you think about the distribution of MNP?

Supplement to: Brewer JD, Shinnick J, Román K, Santos MP, Paz-Soldan VA, Buttenheim AM. Behavioral economic insights into micronutrient powder use for childhood anemia in Arequipa, Peru. *Glob Health Sci Pract*. 2020;8(4). https://doi.org/10.9745/GHSP-D-20-00078

**Theme**	Questions/points
	What do you think about the actions the state is carrying out to reduce the number of anemia cases? Would you be able to pose some suggestions for improvement?<br>What do you believe the government needs to do so that children don't have anemia?<br>Do you believe that the supplements or "chispitas" are enough or not? Why?
